# The Development, Implementation, and Evaluation of a Racism and Health Disparity Prevention Course for Midwives

**DOI:** 10.1111/jmwh.70011

**Published:** 2025-08-13

**Authors:** Arielle E. Skalisky, Melissa A. Saftner

**Affiliations:** ^1^ University of Minnesota School of Nursing Minneapolis Minnesota

**Keywords:** antiracism, curriculum, development, midwifery

## Abstract

Maternal health disparities persist as a significant issue in the United States, with Black, Indigenous, and marginalized women facing disproportionately high rates of maternal morbidity and mortality. Despite the United States having the highest maternal mortality rate among developed countries, addressing these disparities remains a challenge, particularly in rural areas and within underserved communities. One key strategy in mitigating these disparities involves training health care providers to recognize and combat systemic biases, particularly those rooted in race. This article discusses the development, implementation, and evaluation of a dedicated antiracism and health disparity prevention course developed for midwifery students at the University of Minnesota. In addition to content embedded in the existing midwifery curriculum, a course was designed and integrated that addresses racism, health disparities, and cultural competence designed for second‐semester Doctor of Nursing Practice students. Faculty use a structured framework to increase awareness of implicit biases, historical oppression, and the importance of culturally sensitive care. The course encourages self‐reflection and equips students with actionable strategies to address systemic inequities in maternal care. Students reported increased self‐awareness and a stronger commitment to addressing racism in health care. Despite challenges such as balancing credit loads and emotional labor, midwifery students report that it positively contributes to their education. Additionally, the course has garnered interest from students and faculty in other health care disciplines. The authors advocate for the integration of such courses across health care programs to foster more equitable, antiracist practices within maternal health care.

## INTRODUCTION

Maternal health disparities in the United States remain a significant issue and highlight the gaps in health outcomes across racial, socioeconomic, and geographic lines. Globally, the United States has the highest maternal mortality rate among developed countries, with 22.8 deaths per 100,000 live births in 2022.^1^, ^2^ Black and Indigenous women are disproportionately impacted, and Black women are more than twice as likely as White women to die from pregnancy‐related complications.^3^ This disparity can be attributed to systemic factors like implicit bias in health care, socioeconomic status, and reduced access to high‐quality care.^3^ Maternal morbidity and mortality rates are also notably higher in rural areas, where hospital closures and a lack of maternal care specialists compound the risk of poor outcomes.^4^

  

Continuing education (CE) is available for this article. To obtain CE online, please visit http://www.jmwhce.org. A CE form that includes the test questions is available in the print edition of this issue.

Minnesota, although generally regarded as a state with good health care outcomes, reflects similar disparities.^5^ According to the Minnesota Department of Health, compared with White populations, Black pregnant people are 2.3 times more likely to die from pregnancy or childbirth‐related causes and the Indigenous maternal mortality rate is approximately 4 times higher despite making up only 12% to 13% and 2% of the birthing population in Minnesota, respectively.^6^, ^7^

In 2023, Minnesota had 412 midwives certified in the state, with Minneapolis–St Paul having the seventh highest metropolitan employment level of nurse‐midwives in the United States.^8^, ^9^ Demographic data for the Minnesota midwifery workforce are not available, although in 2019, 91% of all advanced practice registered nurses in the state identified as White,^10^ and the Minnesota Hospital Association notes the Black, Indigenous, and person of color (BIPOC) health care workforce has increased by 84% since 2017.^11^ The American Midwifery Certification Board noted that nationally, 83.3% of midwives identified as White.^12^ It is likely that Minnesota's midwifery workforce is less diverse than the national workforce although communities of color make up 20% of the Minnesota population.^13^

**QUICK POINTS**

✦ Addressing maternal health disparities requires education programs to train health care providers to recognize and respond to the unique needs of diverse populations.
✦ In the absence of sufficient diversity within health care professions, it is imperative to train culturally competent, responsive, and equity‐driven providers who can act as effective allies.
✦ A dedicated antiracism course was beneficial to midwifery learners, regardless of racial or ethnic background, previous experience with antiracism content, or lived experience.

Despite the state's robust health care infrastructure, factors such as access to culturally competent care and socioeconomic barriers remain challenges that impact maternal health outcomes for communities of color. Providing care that aligns with patients’ racial and cultural backgrounds is increasingly acknowledged as essential to delivering comprehensive, person‐centered health services and achieving equitable outcomes.^14^ Studies have linked racially concordant care to increased engagement with health systems, enhanced communication between patients and providers, higher satisfaction levels, and a measurable reduction in racial disparities.^15^ Increasing midwifery workforce diversity is a priority that requires long‐ and short‐term strategies. A key factor in addressing maternal health disparities is ensuring that health care providers are trained to recognize and respond to the needs of diverse populations.^16^, ^17^, ^18^

Evidence shows that implicit biases within health care settings can lead to misdiagnosis, undertreatment, and dismissal of concerns, which disproportionately affect BIPOC and other marginalized communities.^19^, ^20^ By equipping providers with antiracism training and strategies to deliver culturally competent care, health care systems can mitigate these biases and build trust with patients.^21^, ^22^ Health care training programs that include antiracism and health disparity content are favorably viewed by participants and increase overall knowledge and ability to address racism in their practicing institutions.^23^, ^24^ Training health care providers to recognize structural racism, address implicit bias, and engage with the social determinants of health is essential to advance equity in maternal care.^25^

This foundational shift in provider education is particularly relevant in Minnesota, where targeted training is crucial to closing the persistent racial disparities in maternal health outcomes. In response to the murder of George Floyd in 2020 and the ongoing impact of the COVID‐19 pandemic on communities across the United States, the midwifery faculty at the University of Minnesota (UMN) expanded their program curriculum to include a dedicated course on antiracism and health disparity prevention. This course built on the existing content already integrated into all midwifery didactic and clinical courses. This article will explore the development, implementation, and evaluation of this curriculum, providing a roadmap for other programs seeking to enhance their focus on antiracism and health disparity prevention.

## CURRICULUM DEVELOPMENT AND IMPLEMENTATION

Up until 2022, UMN's midwifery program included content on antiracism and health disparity prevention in all didactic, core, and clinical midwifery courses. Although the content was available, it was fragmented. The faculty determined that it was necessary to create a course that could inform all future midwifery coursework. The UMN midwifery program director asked midwives from other institutions about similar courses in their universities. At the time of course development, there was one course in existence at a state university on the west coast. The midwifery program director agreed to act as a consultant in the development of the UMN course. The consultant provided guidance on creating the course and for structuring assignments and topics. Additionally, they provided recommendations for considering Minnesota‐specific populations in course creation given the large number of immigrants and refugees living in the state. Existing course content on health disparities and antiracism remained embedded in core and midwifery courses during and after course implementation. Our objectives were not to replace existing content but to ensure antiracist work was woven through the curriculum with a solid foundation in year one.

Working collaboratively with midwifery faculty and the faculty consultant, we developed a course description and objectives. The course description included the statement that it would “prepare students to understand the impact of history, societal structure, systems of oppression, power dynamics and privilege on health care delivery and outcomes, especially in the context of pregnancy and reproduction.” Course objectives were created to bring or increase awareness of the various forms of racism (individual, systemic, and structural)^26^; develop critical thinking through reflection on concepts of privilege, bias, and power dynamics; foster empathy and solidarity; and empower action by providing students with strategies to actively combat racism in their own lives and communities. The course was planned for students in the second semester (of 8 total semesters) enrolled in the Doctor of Nursing Practice program.

The course structure was designed using Sukhera and Watling's framework, which includes 6 key features: point 1: creating a safe and nonthreatening learning context; point 2: increasing knowledge about the science of implicit bias; point 3: emphasizing how implicit bias influences behaviors and patient outcomes; point 4: increasing self‐awareness of existing implicit biases; point 5: improving conscious efforts to overcome implicit bias; and point 6: enhancing awareness of how implicit bias influences others (see Table 1).^27^ The course was structured with 13 weekly content modules, one project work week, and one week for presentation and peer response.

**Table 1:** Examples of Content Modules Guided by the Sukhera and Watling Framework

Sukhera and Watling's Framework	Content Module	Sample Readings or Resources
Points 1, 2	Week 1: Introduction and Course Overview	1. Kirk G, Okazawa‐Rey M. Identities and social locations: who am I? Who are my people? Chapter in *Women's Lives: Multicultural Perspectives*. McGraw Hill; 2007. 2. McIntosh P. *White Privilege: Unpacking the Invisible Knapsack*. Peace and Freedom;1989. 3. Oluo I. Introduction in *So You Want to Talk About Race*. Seal Press; 2018: 1‐7.
Points 3, 4, 6	Week 3: Introduction to Racism and Health Care	1. Eichelberger KY, Doll K, Ekpo GE, Zerden ML. Black Lives Matter: claiming a space for evidence‐based outrage in obstetrics and gynecology. *Am J Public Health*. 2016;106(10):1771‐1772. 2. Hardeman RR, Medina EM, Kozhimannil KB. Structural racism and supporting Black lives ‐ the role of health professionals. *N Engl J Med*. 2016;375(22):2113‐2115. 3. Kenen J, Bachelor E. Racist doctors and organ thieves: why so many Black people distrust the health care system. *Politico*. December 18, 2022. https://www.politico.com/news/magazine/2022/12/18/black‐mistrust‐healthcare‐00060324
Point 6	Week 9: History of Racism in Midwifery	1. *Truth and Reconciliation Resolution from the American College of Nurse‐Midwives*. ACNM; 2021. https://midwife.org/wp-content/uploads/2024/10/ACNM-Truth-and-Reconciliation-Resolution.pdf. 2. Jumah NA, et al. On the path to reclaiming Indigenous midwifery: co‐creating the Maternal Infant Support Worker pilot program. *Int J Gynecol Obstet*. 2021;155(2):203‐210. 3. McCouch, H. Navajo nurse midwife brings healthcare to women and mothers of Standing Rock. Medium. 2016. https://medium.com/every‐mother‐counts/from‐a‐navajo‐nurse‐midwife‐what‐you‐should‐know‐about‐the‐women‐and‐mothers‐of‐standing‐rock‐8aea263734aa 4. Niles PM, Drew M. Constructing the modern American midwife: white supremacy and White feminism collide. Nursing Clio. October 22, 2020. https://nursingclio.org/2020/10/22/constructing‐the‐modern‐american‐midwife‐white‐supremacy‐and‐white‐feminism‐collide
Point 5, 6	Week 11: Workforce Diversity	1. Goode KL. “Sick and tired of being sick and tired”: situating midwifery within a womanist ethic of caring justice. Chapter 5 In: Birthing, Blackness, and the Body: Black Midwives and Experiential Continuities of Institutional Racism PhD dissertation. CUNY University of New York; 2014: 147‐172. 2. Marcinko T. More Native American doctors needed to reduce health disparities in their communities. American Association of Medical Colleges. November 13, 2016. http://www.aamc.org/news/more-native-american-doctors-needed-reduce-health-disparities-their-communities 3. Serbin JW, Donnelly E. The impact of racism and midwifery's lack of racial diversity: a literature review. *J Midwifery Womens Health*. 2016;61(6): 694‐706.

Source: Sukhera and Watling.^27^

Each module was structured uniformly and included 2 to 4 unique learning objectives that set clear outcome expectations. A checklist of action items for the week along with a list of required and recommended readings and resources was also provided. Readings and resources were curated to align with objectives and engage students at various levels of understanding about the module topic. Resources included book chapters, peer‐reviewed research articles, news and opinion pieces, policy documents, and multimedia resources (videos, websites, etc) with the goal of offering multiple viewpoints from diverse perspectives on the topic (see Table 1). Students were encouraged to share any additional resources encountered while completing module work in a separate, open forum on a discussion board in their online course platform.

In the first half of the semester, students completed weekly self‐reflections on the module topics. Students were asked to identify new concepts or existing concepts that stood out to them, express any feelings that came up when working through the module content, and connect the information to their intentions as future clinicians (see Table 2). Assignments for the middle semester modules included group discussions initiated by a question prompt. Students were expected to answer the question prompt individually, citing sources from the weekly content to support their response and then engage in discussion with their peers throughout the week. In one of the final weeks, students were asked to write a 3‐ to 4‐page article reflecting on their key takeaways from the course.

**Table 2:** Examples of Weekly Assignments

Module	Module Objectives	Assignment	Prompt
Week 4: Introduction to Understanding Bias	1. Define microaggressions and stereotype threat. Review definition of implicit bias. 2. Understand how racism, implicit bias, and microaggressions can play out in a health care setting (how one delivers or receives health care). 3. Understand the role of stereotype threat on learning and patient care.	Self‐reflection	1. What is/are your bias(es)? How do they impact the care you provide as a nurse? What do you need to do to ensure that your biases don't cause harm to others? 2. What did I learn that was new in the content? Why would this be important to me in my practice as a midwife? 3. Did anything make me uncomfortable or angry while I was reading today? How did I work through that discomfort? Why does it matter that it made me uncomfortable or angry?
Week 6: Impact of Colonialism on Global Health	1. Define the concepts of globalization and colonialism. 2. Describe how colonialism has historically impacted global health 3. Describe how technology and politics contributed to global health inequities	Self‐reflection	1. What are examples of colonialist practices in Global Health? How do midwives in the Global North ensure an anticolonialist approach in work with colleagues? Are there any examples of ethical global health endeavors? What makes them ethical? 2. What did I learn that was new in the readings? Why would this be important to me in my practice as a midwife?
Week 9: History of Racism in Midwifery	1. Describe the history of BIPOC and immigrant midwives in the United States. 2. Describe the history of the American College of Nurse‐Midwives and the ACNM Midwives of Color Committee. 3. Describe current efforts within midwifery professions to overcome racism and racial bias.	Group Discussion	1. Review/share 2‐3 of the most startling statistics or disparities you've come to understand in the first 7 weeks of this course. 2. What do you think you can do on the micro and macro level to eliminate racism within the midwifery community? Be specific. 3. How has ACNM addressed racism within the organization in recent years? Do you think they have done a good job of addressing racism as a professional organization? Why or why not?

Abbreviations: ACNM, American College of Nurse‐Midwives; BIPOC, Black, Indigenous people of color.

In addition to the online modules, there were 4 synchronous, virtual class sessions. The purpose of the synchronous sessions was to facilitate real‐time interaction, discussion, and engagement with students around course content in a safe and open forum. Guest speakers with unique perspectives were invited to join the virtual classes. For example, a physician colleague presented on the history of racism in obstetrics and gynecology with special attention to gynecologic procedures, such as fistula repair technique, that were perfected on enslaved persons.^28^

In the final 2 weeks of the course, students were tasked with illustrating an advocacy‐based partnership to act against systemic, interpersonal and internalized racism. This was accomplished through a group project where students chose a health disparity and proposed a new social policy or changes to an existing one to address the disparity. They were required to include (1) the intended impact of the policy, (2) who would be most impacted, (3) potential unintended consequences, (4) a description of a community‐based or national organization currently working to address the disparity, and (5) the role of individual midwives and nurse practitioners in addressing the disparity. Examples of topics covered in the final project include prenatal care access for undocumented immigrants, addressing maternal mental health care in the United States, and the shackling of pregnant and incarcerated women. In the final weeks of the session, students were asked to write a 3‐ to 4‐page article reflecting on their key takeaways from the course.

### Barriers to Implementation

There were barriers encountered during the development of the course. Initially, the School of Nursing's curriculum committee raised concerns over adding credits to the already high credit load for midwifery students. However, the value of the course content outweighed the concerns for an increased workload or increase in tuition. UMN allows students to bundle tuition. Therefore, as most students were already enrolled in 9 credits, there would be no additional increase in tuition with the added credits.

Although the course was developed strategically to balance the workload students experienced in their first year of the program, the faculty understood that the emotional toll of processing the course content could be difficult to account for. Self‐care strategies, including verbal check‐ins and centering exercises, were built into the class and reviewed during each synchronous session. Additionally, some faculty members expressed concerns about how to best support students in engaging with challenging topics without overwhelming them. To address this, the course was designed to incorporate regular debriefing sessions and opportunities for reflective practice. Faculty had the opportunity to participate in a free, university‐offered seminar on navigating challenging conversations that was offered by the Office of Equity and Diversity. Faculty participated in training to ensure they were equipped to facilitate difficult conversations effectively. These steps helped foster a supportive learning environment that emphasized growth and resilience.

The course was designed by 2 midwives: the program director, a White cisgender woman, and an adjunct faculty member, a Black cisgender woman. Given the adjunct faculty member's passion for the topic and overlapping work in the community, it was determined that she would be the lead faculty for the course. The faculty also agreed that having the course taught by a BIPOC faculty member could be most supportive of all student learning. That is, it allowed both BIPOC and non‐BIPOC students to have a faculty expert and engage with the instructor in different ways. Given the low number of BIPOC midwives nationally, this option may not be feasible in all institutions.

## COURSE EVALUATION

**Figure 1:**
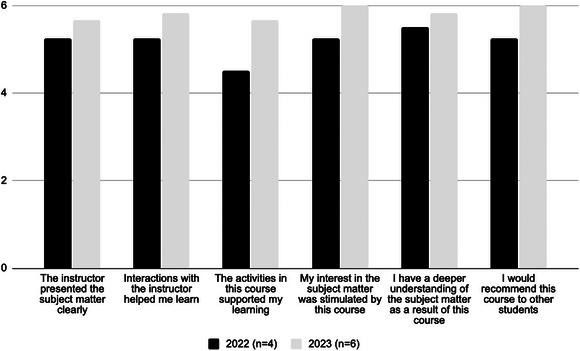
Student Course Evaluation Ratings

The course was launched January 2022 as a one‐credit, graded course restricted to first year midwifery students. In total, 25 students have successfully completed the course from 2022 to 2024. At the end of each semester, students completed anonymous evaluations seeking instructor and course feedback and suggestions. The first 2 years of the course, slightly more than half (n = 10) completed the course evaluations (see Figure 1). Unfortunately, the UMN minimum threshold was not met for the 2024 cohort, so no evaluation data are available. However, despite the low number of evaluations, students reported an overall positive experience with the course, and a majority would recommend this course to others.

As part of the course evaluation process, students are encouraged to provide open‐ended feedback about the course at the end of the evaluation. This area of evaluation was particularly helpful in understanding the impact of the course on students and garner information about what students desired in the future (see Table 3). Student comments highlighted how the course provided an opportunity for them to consider their own privilege. It also allowed students to take time to consider their own bias and develop systems to counteract those biases. Additionally, students appreciated how the instructor provided ongoing feedback and requested more opportunities to meet synchronously. Students underscored the importance of having a midwife of color teach the course. This intentional faculty assignment allowed both BIPOC and White students to build a safe space for exploration and discovery.

**Table 3:** Student Qualitative Feedback on Course Evaluations

Feedback from Students
*“I've appreciated the opportunity to examine my own understandings and slowly write them, sit in them, edit them, and reflect on them. I find that I am developing the skill of reflection without reaction, which is necessary as a White woman recognizing my role in this system of oppression.”*
*“[the instructor] promoted a respectful and open environment to discuss vulnerable topics as well as gave good insight and feedback on my personal reflections.”*
*“I would like there to be more Zoom class sessions to discuss the topics as a group. In this, we can learn more from the instructor, her experiences, and her thoughts.”*
*“Expand this course to other specialties.”*
*“[the instructor] provided a SAFE and uncharted territory for her BIPOC and White students alike. There was never a moment where I did not feel like I couldn't share my honest thoughts and feelings about large systemic issues while also expanding upon my learning about racism and midwifery as a profession. This class NEEDS to be streamlined everywhere.”*
*“Overall, this course was great. I know there is talk about increasing credits, which would be great to provide more education and ability to discuss this information with others.”*

Abbreviation: BIPOC, Black, Indigenous people of color.

Students who identified as student midwives of color also found the course to be useful to their professional growth. Comments from students emphasized the importance of the course and the need to train all health care professionals in antiracism. Additionally, feedback from students included a request for more content. Students wanted more information about the history and politics of reproductive health in addition to more time to explore the local and global context of antiracism and health disparity prevention in the course.

Given the positive overall course reviews, the faculty determined that it would be prudent to increase the course to 2 credits to allow more time for students to engage in the course content and explore the history and current landscape of health disparities. Course revisions and updates were proposed through the school's curriculum committee, and having the student data and feedback allowed the midwifery faculty to increase the credit load with ease. Finally, although not a direct result of the course evaluation process, since implementation of the course, midwifery applicants to the program have highlighted the course as a reason they were applying to the UMN midwifery program.

## DISCUSSION

The development and implementation of this course was in response to the increasing demand for midwives, as direct care providers, to have foundational knowledge of racism, health and health care disparities, and an introduction to strategies for antiracist efforts.^7^, ^8^, ^9^ Providing a dedicated course for midwifery students allows for systematic dissemination of critical history, such as medical experimentation on Black bodies and the transparent history of midwifery, while also providing a safe space for self‐reflection and processing of this information. Course faculty recognize that all individuals are at different stages of their journey to understand the history and ongoing impacts of racism in the United States.

Building a midwifery workforce that is knowledgeable about the true history of the profession is imperative to mitigating the risk of repeating past mistakes. Fostering growth of equity‐minded, culturally sensitive midwives promotes reduction and hopeful elimination of health and health care disparities in reproductiveand sexual health. The dismantling of systemic racism in health care requires health care providers to identify and address their implicit biases, build rapport with communities they serve, and advocate for sustainable change in social policies.

It is the belief of the authors that this course should be done in concordance with strategies and efforts to recruit and retain midwifery students from underrepresented racial and ethnic backgrounds to promote a profession that reflects the communities it serves. Ultimately, although it does not guarantee cultural competence or responsiveness, the provision of racially concordant care may contribute to the creation of a validating and empowering environment for communities to engage in health care.^15^

### Implications for Practice

The profession of midwifery has remained largely homogenous, with 83% of certified nurse‐midwives/certified midwives identifying as White.^12^ This lack of diversity stands in stark contrast to the increasing disparities in maternal morbidity and mortality among BIPOC individuals, underscoring the urgent need for racially and culturally concordant care to improve health outcomes.^22^, ^29^, ^30^ In the absence of sufficient diversity within the profession, it is imperative to prioritize the training of culturally competent, responsive, and equity‐driven health care providers who can act as effective allies.^21^, ^26^, ^31^ These providers must be equipped to recognize systemic inequities, address biases, and deliver care that respects and centers the lived experiences of diverse populations.

### Implications for Education

On April 23, 2025, an executive order was signed that limited accrediting bodies’ ability to require diversity initiatives as part of accreditation reviews.^32^ On April 24, 2025, the Accreditation Commission for Midwifery Education distributed information to midwifery programs highlighting changes to the language required for accrediting, likely to meet the intent of the executive order. These executive orders could have significant ramifications for antiracist pedagogy. Given this change, courses like the one described in this article are even more critical to ensuring that students are trained to in antiracism and learn strategies to mitigate health disparities.

### Lessons Learned

The authors acknowledge that the volume of feedback received from course participants was limited. Despite the small number of responses, the authors have received numerous inquiries from students in other advanced practice registered nurse specialties, as well as from nonnursing disciplines, expressing interest in accessing or adapting the course for broader use among health care professionals in training. Although the authors advocate for antiracism education to be seamlessly integrated throughout the curriculum, they recognize the distinct value of a dedicated course tailored to midwifery students. Feedback and engagement since the course's inception underscore its importance in fostering critical reflection and promoting equity in practice, making a strong case for its broader dissemination and interprofessional applicability.

## CONCLUSION

Designing and implementing an antiracism course is not just about imparting knowledge; it is about inspiring a mindset shift and equipping people to act. The content should foster empathy, self‐reflection, and responsibility and help participants understand their role in creating a more equitable society. It is an ongoing process that requires continual learning, humility, and commitment to change. By working together, midwives can push toward a health care system that acknowledges past wrongs, confronts present inequities, and actively works toward a more just, inclusive, and compassionate future. This comprehensive approach, rooted in history, advocacy, education, and community trust, can create a truly transformative shift in maternal and reproductive health care, making it more equitable for all.

## CONFLICT OF INTEREST

The authors have no conflicts of interest to disclose.
